# Primary Adrenal Lymphoma Possibly Associated With Epstein–Barr Virus Reactivation Due to Immunosuppression Under Methotrexate Therapy

**DOI:** 10.1097/MD.0000000000001270

**Published:** 2015-08-07

**Authors:** Yu Ohkura, Junichi Shindoh, Shusuke Haruta, Daisuke Kaji, Yasunori Ota, Takeshi Fujii, Masaji Hashimoto, Goro Watanabe, Masamichi Matsuda

**Affiliations:** From the Department of Gastroenterological Surgery, Hepato Pancreato Billiary Surgery Unit (YO, JS, SH, MH, GW, MM) and Departments of Hematology (DK) and Pathology (YO, TF), Toranomon Hospital, Tokyo, Japan; and Isetan Mitsukoshi Ltd., Nihonbashi Mitsukoshi Main Store, Tokyo, Japan (MM).

## Abstract

Primary adrenal lymphoproliferative disorder (LPD) is an extremely rare disease that is widely known to be associated with methotrexate (MTX) use in patients with rheumatoid arthritis (RA).

A 70-year-old man was incidentally found to have a tumor at the dorsal part of the liver in a medical check-up. He had a history of RA treated with MTX. Abdominal ultrasonography demonstrated a low echoic mass (30 mm in diameter) at the dorsal part of the liver, located close to the inferior vena cava. Preoperative differential diagnoses included intrahepatic cholangiocarcinoma, adrenal tumor, and hepatic malignant lymphoma, but no definitive diagnosis was reached. On exploratory laparotomy, the tumor seemed to be derived from the right adrenal gland and adhered tightly to segment 7 of the liver. Therefore, right adrenectomy with partial resection of segment 7 of the liver was performed. Pathological findings revealed diffuse inflammatory cell infiltration with a population of small atypical lymphoid cells, with positive immunohistochemical evidence for Epstein–Barr virus (EBV). Final diagnosis was primary adrenal iatrogenic EBV-positive LPD, classified as “other iatrogenic immunodeficiency-associated LPDs: Hodgkin-like lesions.”

In this report, we described the possibility of the spontaneous healing of MTX-associated LPD (MTX-LPD) before treatment and the importance of doubting MTX-LPD and doing immunostaining to necrotic tissue. To our knowledge, this is the first reported case of MTX-related EBV-positive LPD, Hodgkin-like lesion, of the unilateral adrenal gland in patient with RA.

## INTRODUCTION

Primary extranodal lymphomas account for approximately 1/3 of all lymphomas and can affect almost any organ, most commonly the skin and stomach, followed by the thyroid, bone marrow, lung, and so forth.^1^ The adrenal gland is often the site of metastasis from primary lung, breast, kidney, bladder, pancreas, and skin cancer.^2^ However, primary adrenal lymphoproliferative disorder (LPD) is an extremely rare extranodal lymphoma that is characterized by a high incidence of bilateral involvement and predominantly diffuse large B-cell histology.^2–4^ LPD is widely known to be associated with methotrexate (MTX) use in patients with rheumatoid arthritis (RA).^5–8^ And also past reports have described LPD associated with Epstein–Barr virus (EBV),^9^ but not of primary adrenal MTX-related EBV-positive LPD. The first choice of treatment strategy for MTX-associated LPD (MTX-LPD) is typically discontinuation or dose reduction of MTX. However, the pathogenesis of MTX-LPD still remains unclear and the treatment of MTX-LPD has been controversial. This case suggests the one important possibility for considering the mechanism or treatment of MTX-LPD. This report was approved by our hospital's Institutional Review Board results.

## CASE REPORT

A 70-year-old man who was incidentally found to have a liver tumor on enhanced computed tomography (CT) during a comprehensive medical examination was referred to our hospital in April 2014. He was diagnosed with RA in 1994 and MTX had been administered since 2006 at a dose of 12 mg/week.

**FIGURE 1:**
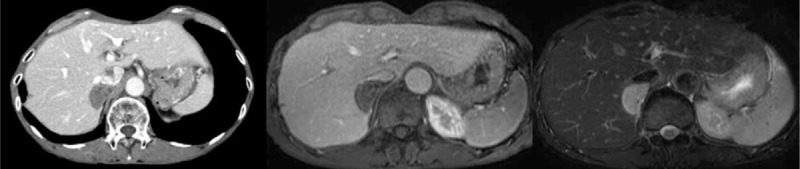
The tumor (approximately 40 mm in diameter) was hypovascular on enhanced computed tomography scan (right), indicated low intensity on T1-weighted MRI (center), and high intensity on T2-weighted or diffusion MRI (left). Dynamic study revealed peripheral enhancement on a late phase. The tumor located close to the inferior vena cava. MRI = magnetic resonance imaging.

His family history was unremarkable. Physical examination revealed no specific abdominal findings. The results of routine blood examination and liver function tests were normal, and serum tumor markers (CEA, CA19-9, DUPAN-2, and Span-1) were within normal limits. Serum cortisol was 14.1 μg/dL (normal: 4.5–21.1 μg/dL) and corticotropin was 91.7 pg/mL (normal: 7.2–63.3 pg/mL). Serum epinephrine was 0.02 ng/mL (normal: ≤0.1 ng/mL), norepinephrine was 0.048 ng/mL (normal: 0.1–0.45 ng/mL), and dopamine was 0.02 ng/mL (normal: ≤0.02 ng/mL). Abdominal ultrasonography demonstrated a low echoic mass (30 mm in diameter) at the dorsal part of the liver adjacent to the inferior vena cava (IVC). The tumor was hypovascular on enhanced CT scan (Figure 1) and indicated low intensity on T1-weighted magnetic resonance imaging (MRI) and high intensity on T2-weighted or diffusion MRI (Figure 1). Dynamic study revealed peripheral enhancement on a late phase.

**FIGURE 2:**
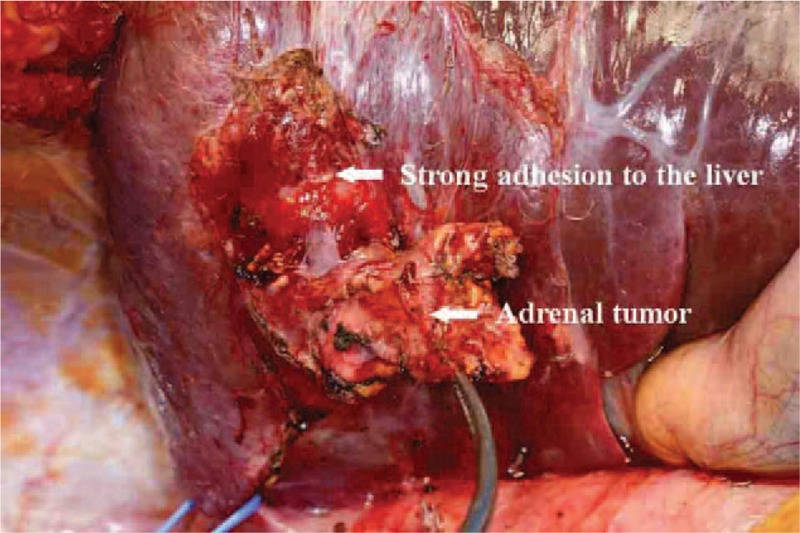
Intraoperative findings showing the tumor located right dorsal to the IVC and derived from the right adrenal gland. The tumor showed strong adhesion to hepatic segment 7. Right adrenectomy was performed extending to segment 7 of the liver. IVC = inferior vena cava.

Preoperative differential diagnoses included intrahepatic cholangiocarcinoma, adrenal tumor, and hepatic malignant lymphoma; however, no definitive diagnosis could be reached. We therefore performed exploratory laparotomy. Intraoperative finding revealed a right adrenal gland mass with direct infiltration to segment 7 of the liver (Figure 2), and right adrenectomy was performed extending to segment 7 of the liver. Because we had not reached definite diagnosis before operation, we had thought to diagnose due to biopsy during operation as 1 choice. However, we performed complete resection of the tumor as excisional biopsy under the condition that we could not find another malignancy around the origin from preoperative imaging or intraoperative findings. We did not think the need of extra resection whatever the results came out because we performed complete resection of the tumor, so we did not perform pathological diagnosis during surgery.

**FIGURE 3:**
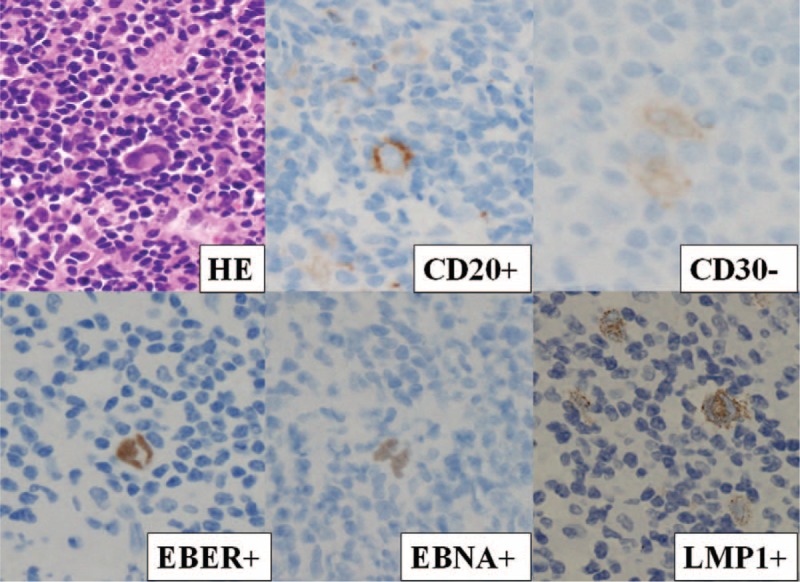
Atypical lymphocytes were confirmed by strongly positive immunohistochemical staining for CD20, EBER, EBNA, and LMP1 but negative staining for CD30 (×60). This confirmed the diagnosis of primary adrenal methotrexate-associated Epstein–Barr virus-positive B-cell lymphoproliferative disorder. EBER = EBV-encoded RNA transcript; EBNA = Epstein–Barr nuclear antigen; LMP1 = latent membrane protein 1; LPD = lymphoproliferative disorder.

**FIGURE 4:**
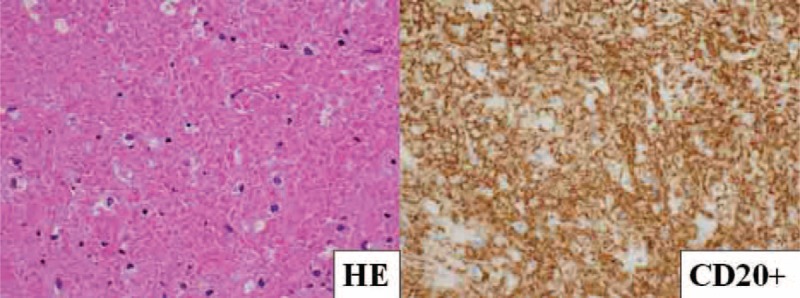
Necrotic tissue was strongly positive for CD20 (×40). The adrenal tumor showed characteristic changes, with predominant geographic necrotic tissue and diffuse inflammatory cell infiltration in the center of the tumor. Since necrotic tissue tends to contain few tumor cells, it can be extremely difficult to accurately diagnose a tumor, and hematoxylin–eosin staining alone fails to provide a definitive diagnosis.

Histopathologically, the adrenal tumor showed diffuse inflammatory cell infiltration with a population of small atypical lymphoid cells. Because the infiltration extended to the liver, the boundary between the liver and adrenal grand tissues was ambiguous in some resected portions. The center of the tumor showed geographic necrotic tissue with diffuse inflammatory cell infiltration. In the necrotic tissue, cellular outlines resembling lymphocytes were noted, indicative of lymphoma. The presence of atypical lymphocytes was confirmed by strongly positive immunohistochemical staining for CD20, EBV-encoded RNA transcript (EBER), Epstein–Barr nuclear antigen (EBNA), and latent membrane protein 1 (LMP1). Staining was negative for CD2, CD3, CD4, CD5, CD7, CD8, CD10, CD15, CD21, CD30, CD56, Bcl-1, Bcl-2, Bcl-6, and MUM1 (Figure 3). The geographic necrotic tissue was strongly positive for CD20 (Figure 4). Immunohistochemistry using the mouse monoclonal antibodies against human CD20 (1:5 dilution; Dako Japan, Tokyo, Japan, clone L26), EBNA (1:100 dilution; Abcam, Cambridge, UK, clone PE2), and LMP1 (1:1000 dilution; Dako Japan, clone CS.1–4), as well as EBER in situ hybridization were performed on formalin-fixed paraffin-embedded tissue sections using an BenchMark GX automated staining instrument (Ventana, Tucson, AZ) according to the manufacturer's instruction.

Given these findings, the final pathologic diagnosis was primary adrenal MTX-associated EBV-positive B-cell LPD, classified as “other iatrogenic immunodeficiency-associated LPDs; Hodgkin-like lesions” by the World Health Organization (WHO).^10^ The additional blood test revealed positive for serum anti-EBV [viral capsid natigen (VCA)] IgG antibody, anti-EBNA antibody, and negative for anti-EBV (VCA) IgM antibody.

Postoperatively, we performed whole-body gallium scintigraphy as an additional investigation and no hot spots were detected. LPD was stage IE according to the Ann Arbor classification.^11^ MTX therapy for RA was discontinued and the patient was followed regularly. He has remained relapse-free for almost 1 year.

## DISCUSSION

Primary malignant lymphoma of the adrenal gland is a rare and highly aggressive disease that has been reported only rarely in the literature.^1–4^ It represents only 3% of extranodal lymphomas.^3^ Secondary involvement of the adrenal gland with non-Hodgkin's lymphoma occurs more frequently, in nearly 25% of patients during the course of disease.^9^ Secondary adrenal involvement generally affects elderly individuals, especially those with a history of cancer, human immunodeficiency virus infection, or autoimmune disorder, and in about 50% of cases it manifests as adrenal insufficiency due to bilateral adrenal involvement.^12^

Rashidi and Fisher^4^ defined primary adrenal lymphoma as a histologically proven lymphoma that involves 1 or both adrenal glands and has at presentation no prior history of lymphoma elsewhere and unequivocally dominant adrenal lesions in cases with lymph nodes or other organ involvement. In our case, preoperative enhanced CT revealed no other lesions except that in the right adrenal gland and postoperative whole-body gallium scintigraphy showed no hot spots. The tumor involved hepatic segment 7, but the adrenal lesion was unequivocally dominant. Accordingly, we diagnosed unilateral primary adrenal LPD.

In 1991, Ellman et al^5^ first reported the association between lymphoma and MTX therapy in patients with RA. Patients with RA have various immune abnormalities, including T-cell dysfunction and B-cell activation associated with autoantigen stimulation.^6^ Thought the pathogenesis of RA-associated lymphoma remains unclear, it has been speculated that high inflammatory activity associated with RA, immunosuppressive agents including MTX used for treatment, or EBV infection/reactivation.

Actual risk elevation for lymphoma among patients treated with MTX is unclear. However, given the reported fact that there were several cases with LPD regressed after discontinuation of MTX therapy,^7^ MTX itself might have strong correlation with LPD in some cases. In addition, it is well known that immunosuppression predisposes patients to EBV-related lymphomas. Suzuki et al^8^ reported that the developmental mechanism of MTX-LPD may include reactivation and/or persistence of EBV due to immunosuppression by MTX. Indeed, EBV infection was detected in 12% to 44% of lymphomas emerged in patients with RA.^7^

In current case, blood test revealed positive result for serum anti-EBV (VCA) IgG antibody, anti-EBNA antibody and negative for anti-EBV (VCA) IgM antibody. In addition, the atypical lymphocytes was confirmed in pathologic specimen by strongly positive results in immunohistochemical staining for EBER and EBNA. There results suggest that chronic viremia due to reactivation of EBV may have played a strong role in development of lymphoma in the current case. Given the reported evidence, MTX usage might have relation with reactivation of EBV in this case.

Iatrogenic immunodeficiency-related LPD usually regresses upon withdrawal of the drug; it is thus considered attributable to a toxic/metabolic effect rather than being EBV-related. In a previous study, tumor size decreased in 20% to 30% of cases of MTX-LPD following the discontinuation of MTX.^6^ The first choice of treatment strategy for MTX-LPD is typically discontinuation or dose reduction of MTX. However, if tumor size does not decrease after discontinuation, chemotherapy or radiotherapy should be performed as second-line therapy. Kawakami et al reported that chemotherapy as the first-line treatment without discontinuation of MTX did not always achieve good outcomes, as infections could occur as a complication.^13^ They recommended starting chemotherapy after stopping MTX whenever possible. Recent descriptions of relapsed Hodgkin's lymphoma that were EBV-positive at initial diagnosis but EBV-negative at recurrence raise the possibility that DNA is lost during tumor progression in some individuals.^14,15^ Salloum et al reported that 62.5% of patients observed after MTX withdrawal without additional antitumor therapy showed at least partial regression in response, and most of these patients were EBV-positive.^16^ Further, survival ratios are higher in patients with Hodgkin's lymphoma than in those with Hodgkin-like lesions.^17^

Histopathologically, atypical lymphocytes were confirmed by strongly positive immunohistochemical staining for CD20, EBER, EBNA, and LMP1. This confirmed the diagnosis of primary adrenal MTX-associated EBV-positive B-cell LPD. In our case, the adrenal tumor showed characteristic changes, with predominant geographic necrotic tissue and diffuse inflammatory cell infiltration in the center of the tumor. Typically, MTX-LPD is not accompanied by tumor necrosis. Since necrotic tissue tends to contain few tumor cells, it can be extremely difficult to accurately diagnose a tumor, and hematoxylin–eosin staining alone fails to provide a definitive diagnosis. In the present case, although the primary adrenal tumor contained a mostly necrotic center, immunostaining of tissue assisted in the diagnosis. In the necrotic tissue, the cellular outlines of lymphocytes indicated lymphoma. Further, immunostaining revealed that the necrotic tissue was strongly positive for CD20.

Generally, such necrosis is attributed to the efficacy of chemotherapy or another treatment. It was generally regarded as MTX withdrawal will restore antitumor immunity and leads the tumor cell necrosis. However, our patient had not received treatment for the adrenal tumor and so we have no identifiable etiology for the tumor necrosis. In other words, the tumor cell might be healing spontaneously in spite of the presence or absence of MTX withdrawal in the past reports. There was no report in the past that the MTX-LPD became necrotic without treatment. And also, it is extremely difficult to diagnose the necrotic tissue like this case by the normal pathological examination. However, if we will be able to doubt MTX-LPD, we can add to do immunostaining and provide definitive diagnosis.

## CONCLUSION

Here, we have reported the case of primary unilateral adrenal iatrogenic MTX-associated EBV-positive LPD with Hodgkin-like lesions.
